# Langerhans Cells From Mice at Birth Express Endocytic- and Pattern Recognition-Receptors, Migrate to Draining Lymph Nodes Ferrying Antigen and Activate Neonatal T Cells *in vivo*

**DOI:** 10.3389/fimmu.2020.00744

**Published:** 2020-04-27

**Authors:** Miguel Angel Becerril-García, Juan Carlos Yam-Puc, Raúl Maqueda-Alfaro, Nonantzin Beristain-Covarrubias, Monica Heras-Chavarría, Isis Amara Gallegos-Hernández, Juana Calderón-Amador, Rosario Munguía-Fuentes, Luis Donis-Maturano, Adriana Flores-Langarica, Leopoldo Flores-Romo

**Affiliations:** ^1^Department of Cell Biology, Center for Advanced Research, The National Polytechnic Institute, Cinvestav-IPN, Mexico City, Mexico; ^2^Institute of Immunology and Immunotherapy, College of Medical and Dental Sciences, University of Birmingham, Birmingham, United Kingdom

**Keywords:** neonates, immunization at birth, T cell activation, *in vivo* immune response, CD204, CD14, TLR-4

## Abstract

Antigen capturing at the periphery is one of the earliest, crucial functions of antigen-presenting cells (APCs) to initiate immune responses. Langerhans cells (LCs), the epidermal APCs migrate to draining lymph nodes (DLNs) upon acquiring antigens. An arsenal of endocytic molecules is available to this end, including lectins and pathogen recognition receptors (PRRs). However, cutaneous LCs are poorly defined in the early neonatal period. We assessed endocytic molecules expression *in situ*: Mannose (CD206)-, Scavenger (SRA/CD204)-, Complement (CD2l, CDllb)-, and Fc-Receptors (CD16/32, CD23) as well as CD1d, CD14, CD205, Langerin (CD207), MHCII, and TLR4 in unperturbed epidermal LCs from both adult and early neonatal mice. As most of these markers were negative at birth (day 0), LC presence was revealed with the conspicuous, epidermal LC-restricted ADPase (and confirmed with CD45) staining detecting that they were as numerous as adult ones. Unexpectedly, most LCs at day 0 expressed CD14 and CD204 while very few were MHCII+ and TLR4+. In contrast, adult LCs lacked all these markers except Langerin, CD205, CD11b, MHCII and TLR4. Intriguingly, the CD204+ and CD14+ LCs predominant at day 0, apparently disappeared by day 4. Upon cutaneous FITC application, LCs were reduced in the skin and a CD204+MHCII+FITC+ population with high levels of CD86 subsequently appeared in DLNs, with a concomitant increased percentage of CD3+CD69+ T cells, strongly suggesting that neonatal LCs were able both to ferry the cutaneous antigen into DLNs and to activate neonatal T cells *in vivo*. Cell cycle analysis indicated that neonatal T cells in DLNs responded with proliferation. Our study reveals that epidermal LCs are present at birth, but their repertoire of endocytic molecules and PRRs differs to that of adult ones. We believe this to be the first description of CDl4, CD204 and TLR4 in neonatal epidermal LCs *in situ*. Newborns’ LCs express molecules to detect antigens during early postnatal periods, are able to take up local antigens and to ferry them into DLNs conveying the information to responsive neonatal T cells.

## Introduction

Early stages of life are related to high susceptibility to infections, which has been attributed to an immature or ineffective immune system, however, the scarce available research on the immunological competence of newborns is frequently contradictory (1). While most studies in neonates deal with adaptive immunity, reports on cells of innate responses are scarce (1). Quantitative and qualitative differences are involved but the exact mechanisms responsible of such putative immaturity during the neonatal period are not well understood. Murine and human neonatal lymphocytes are functionally different from adults and it is generally accepted that T cells in neonates are biased to a Th2 cytokine profile (2–4). However, it has been shown also that under adequate stimulation, early neonates are competent to mount adult-like adaptive immune responses (5–8). There are crucial factors that in early life can determine either dampened or protective immunity, including the dose of antigen, type of adjuvant and type of cells presenting antigen to naïve T cells (9–11).

The skin is one of the most exposed innate barriers, and likely the first one in being colonized by commensal bacterial right during birth. Many factors impact in the cutaneous immune response, these include the type of birth (vaginal or c- section) as well as the cell subsets that populate its different layers. Langerhans cells and Dermal Dendritic Cells (DDCs) are the main cutaneous APCs subsets with distinctive functions each (12). Langerhans cells are a subset of hemopoietic origin skin resident APCs that form a dense planar network in the epidermis (13). APCs are decorated with a variety of endocytic molecules crucial to implement innate immunity. Some of these molecules referred to as pathogen recognition receptors (PRRs) include scavenger receptors, TLR, C-type lectins, CDl4, mannose receptors and unconventional MHC-related molecules such as members of the CD1 family, among many others (14, 15). LCs and DDC play different roles in the maintenance of cutaneous immunity to foreign antigens and immune tolerance to commensal microbiota and autoantigens (16). LCs display a so called impaired mRNA expression of certain TLRs implicated in bacterial recognition (17). Together with a diminished expression of FcRIIα, a deficiency in processing and presenting bacterial products in a major compatibility complex II (MHCII)-restricted manner has also been described for LCs (18), which may also impact in the type of immune response that LCs might elicit. However, most of these studies have been done in adults (humans and mice) and less is known in the very early stages of life.

It has been suggested that depending on the lineage, maturation stage and activation signals, APCs are able to dictate different types of T cell mediated immune responses or to induce tolerance (19, 20). For instance, it has been shown that LCs play a role in inducing tolerance to skin microbiota through favoring the development of regulatory Foxp3+CD4+ T cells (18). In response to stimuli, LCs become activated, undergo maturation, increase surface expression of MHCII and costimulatory markers such as CD80 and CD86, migrate through lymphatic vessels into the regional DLN where they present processed antigens to T cells, inducing specific immune responses or tolerance for the antigens encountered in the periphery (21, 22).

However, little is known of the age-related maturation and physiology of LCs. Previous works have shown that LCs can be detected before birth in the human epidermis and that developing skin already has HLA-DR+, OKT-6+ (CD1+) and ATPase+ LCs (23, 24). Similar to humans, LCs in rodents derive from precursors that are present in the skin before birth (25, 26), predominantly from embryonic fetal liver (27). Murine neonatal LCs are ATPase+, F4/80+, CD11b+ but lack MHCII and langerin molecules (28). These precursors are recruited and distributed in the epidermis during embryonic stage (embryonic day 18), acquiring MHCII, CD11c and Langerin molecules at birth, proliferating actively during the following week (28–30). However, it is still unclear if LCs are able to respond efficiently during the immediate postnatal period.

In this study, we aimed first at evaluating *in vivo* and *in situ* the expression of several PRRs and endocytic molecules in unperturbed dendritic LCs from early neonate mice (at birth and on subsequent postnatal days), in comparison to their adult counterparts. Our data revealed that LCs in the epidermis of mice at birth are as many as in adult skin. Our subsequent studies showed that, apparently, neonatal LC are not only capable of carrying topically applied antigens into the DLN, but also of contributing to activate neonate T cells to proliferation.

## Materials and Methods

### Animals

Specific pathogen-free BALB/c pregnant female mice were provided by the animal facilities (UPEAL) from the Center for Advanced Research of The National Polytechnic Institute CINVESTAV-IPN. Pregnant mice were kept in separate boxes and continuously observed to be certain of the precise time of birth. Day 0 was considered from birth to 24 h (newborns were used usually within the first 3 h of birth) and the early neonatal period was defined until 7 days. Young mice were considered from 8 days to 5 weeks after birth and at sixth week or older, mice were deemed as adult ones. We assessed mice at 0–7, 12, 21, 30, and 90 days of birth. For histology analysis, at least three newborns and four adult mice were used for each experimental condition, and this was repeated at least three times. Trying to minimize potential variations between the experiments, we used neonatal animals from the same offspring for each experiment performed. For cell suspensions and flow cytometry analysis, lymph nodes from six to eleven pups per offspring were pooled. Animals were sacrificed according to the Animal Use Guidelines for animal care and experimentation of the Institute (UPEAL-CINVESTAV-IPN). The protocol and procedures employed were reviewed and approved by the UPEAL-CINVESTAV Ethics Review Committee.

### Epidermal Sheet Preparation

Adult mice skin was shaved before removal with fine scissors. Then, in both adult and newborn animals, skin was cut in pieces of approximately 1 cm^2^. Subcutaneous fat was carefully removed before placing the skin pieces over previously warmed EDTA 0.5M and incubated for 2 h at 37°C. After this period, epidermis was obtained by firm traction and was carefully stretched. Skin intended for ADPase staining was fixed with paraformaldehyde (PFA)-cacodylate during 2 h. For immunolabelling, fixation was performed in cold acetone during 20 min. After fixation, epidermal sheets were washed with saline solution before processing for the staining procedure.

### Epidermal Langerhans Cell Phenotyping *in situ*

For LC immunolabelling, antibodies to CD1d from Santa Cruz, CD204 (2F8) from Bio-Rad, rat hybridoma supernatants to murine DEC205/CD205 (NLDC-145) and CD207/Langerin (L31) (generous gift from RM Steinman, Rockefeller University, NY), MHCII (kindly provided by Dr. L Santos-Argumedo, CINVESTAV-IPN). CD11b (M1/70), CD14 (rmC5-3), CD16/32 (2.4G2), CD21 (7G6), CD45 (30-F11), and TLR4 (MTS510) from BD Biosciences were applied to small pieces of epidermal sheets overnight at 4°C at optimal dilutions, which were previously determined for each marker. After three washes, secondary biotinylated anti-rat antibodies (Vector Lab, Burlingame CA, United States) were used for 1 h at room temperature. Detection was obtained using streptavidin conjugated to alkaline phosphatase (Dako), or horseradish peroxidase (HRP) (Dako, Santa Clara, CA, United States). Chromogen development was performed with: Fast Red or Fast Blue (Vector Lab, Burlingame, CA, United States) or diaminobenzidine (DAB, Dako, Santa Clara, CA, United States). For CD11b detection an especial fixation procedure was implemented as epidermal sheets were fixed with a mixture of acetone/chloroform (1:1).

To assess the presence of Mannose receptor (CD206), PFA-fixed epidermal sheets were incubated overnight with 100 μg/mL of mannosylated albumin tagged with biotin (Sigma, St Louis, MO, United States), or non-mannosylated albumin tagged with biotin alone as control. Peritoneal macrophages were used as positive controls for the presence of CD204, CD206, TLR4, as well as for CD14 and CD11b.

For ADPase staining epidermal sheets were incubated at 37°C for 1 h in a solution containing 5 mg ADP (Sigma A2754), 21 ml 0.1 M Trismal (Trizma maleate), 0.05 M sucrose, 2.5 ml 5% magnesium sulfate and 0.75 ml 5% lead nitrate. After this incubation, epidermal sheets were washed three times and developed with 0.01% ammonium sulfide in water.

Epidermal sheets were embedded in Shandon Immuno mount (Thermo Scientific, Pittsburgh, PA, United States) on glass slides with the dermal side facing up. Density of cells was scored per area (mm^2^) in at least ten fields for each sample, using at least three different epidermal sheets of the same animal. Results were expressed as the mean ± SD of cells/mm^2^ of epidermis.

### Skin Sensitization

The protocols previously described (31, 32) were used to track the migration of FITC + LCs from skin to DLN. In brief, freshly prepared 0.5% fluorescein isothiocyanate (FITC, Sigma-Aldrich, St Louis, MO, United States) dissolved in 1:1 acetone:dibutylphthalate was painted on newborn trunk (100 μl) and adult shaved abdomen (400 μl). 18 h later, mice were sacrificed and brachial, axillary and inguinal lymph nodes were taken to search for FITC+ cells by flow cytometry of cell suspensions.

### Skin Cryosection

After 18h of FITC sensitization, skin from neonates were immediately placed in optimum cutting temperature (OCT) compound (Leica, Nussloch, Germany) and frozen in liquid nitrogen. Conventional 6 μm skin sections from frozen tissue were stained for ADPase (as described before) and CD204 (2F8, Bio-Rad). Cryosections were fixed with 1% PFA-cacodylate (0.8M)-Sucrose (0.2M) during 30 min or with cold acetone during 20 min, for ADPase or CD204 staining, respectively. For CD204 labeling, a biotinylated antibody was incubated overnight at 4°C at optimal dilution. After three washes, HRP-linked Streptavidin (BD, San Jose, CA, United States) was added for 20 min at room temperature followed by extensive washing. Developing of bound antibodies was achieved using DAB (Dako, Santa Clara, CA, United States) for brown color. Nuclear counterstaining was done with Gill’s hematoxylin.

### Analysis of Antigen Carriage to Lymph Nodes and the Effects on Neonatal T Cells

For FACS analysis, 18 h after cutaneous FITC painting, the DLNs from neonatal or adult mice were dissected, homogenized between the frosted ends of two microscope slides, viability of the cells was determined by trypan blue exclusion. Cells suspensions from these DLNs were analyzed for the presence of antigen-positive cells, for the frequency and the potential activation of T cells and for T cells in different phases of the cell cycle, the latter at 48 and 72 h. Antibodies and reactants used were: antibodies to CD3-PE (145-2C11), CD25-APC (PC61), CD69-PerCP-Cy5.5 (H1.2F3), CD86-PE (GL1), MHCII-PE (M5/114.15.2), CD11c-PE-Cy7 (HL3) from BD Biosciences, CD86-APC (GL1) from Tonbo Biosciences, CD3-BV421 (17A2) from BioLegend, CD204-biotinylated (2F8) from Serotec (Bio-Rad), streptavidin-Alexa-647, and Hoechst 33342 from ThermoFisher (H3570).

Eighteen hours after cutaneous FITC, DLN T cells were assessed both for the frequency and the expression of activation marker CD69. For cell cycle analysis we prepared DLNs cell suspensions 48 and 72 h after topical FITC. We based on the premise that cells in G0-G1 possess a normal diploid chromosomal and hence DNA content (2n) whereas in G2-M contain exactly twice this amount (4n). As DNA is synthesized during S-phase, cells are found with a DNA content ranging between 2n and 4n. A histogram plot of DNA content against cell numbers gives the classical DNA profile for proliferating cells.

Except for the cell cycle, cells were fixed with 1% paraformaldehyde and analyzed in the Fortessa LSRII using the FACS Diva software. Results were analyzed with Flow Jo software v7.6.5 (TreeStar).

### Statistical Analysis

Data on Figure 3 are expressed as mean ± SD and were analyzed for statistical significance by one-way ANOVA-Bonferroni. *p > 0.05, **p > 0.01, ***p > 0.001. Values on Figures 4–6 are dotted and horizontal line indicates the median. Data on Figure 7 represent the mean. Differences between mean values were analyzed for statistical significance with GraphPad Prism 5 Software (La Jolla, CA, United States), using the Mann-Whitney *U* test. In the case of multiple comparisons, Kruskal-Wallis test with the Dunn’s multiple comparisons were used. *P*-values less than 0.05 were considered statistically significant (*P < 0.05; **P < 0.01; ***P < 0.001; ****P < 0.0001).

## Results

### Adult and Newborn Epidermal LCs Differentially Express Various Endocytic Molecules and PRRs *in situ*

**FIGURE 1:**
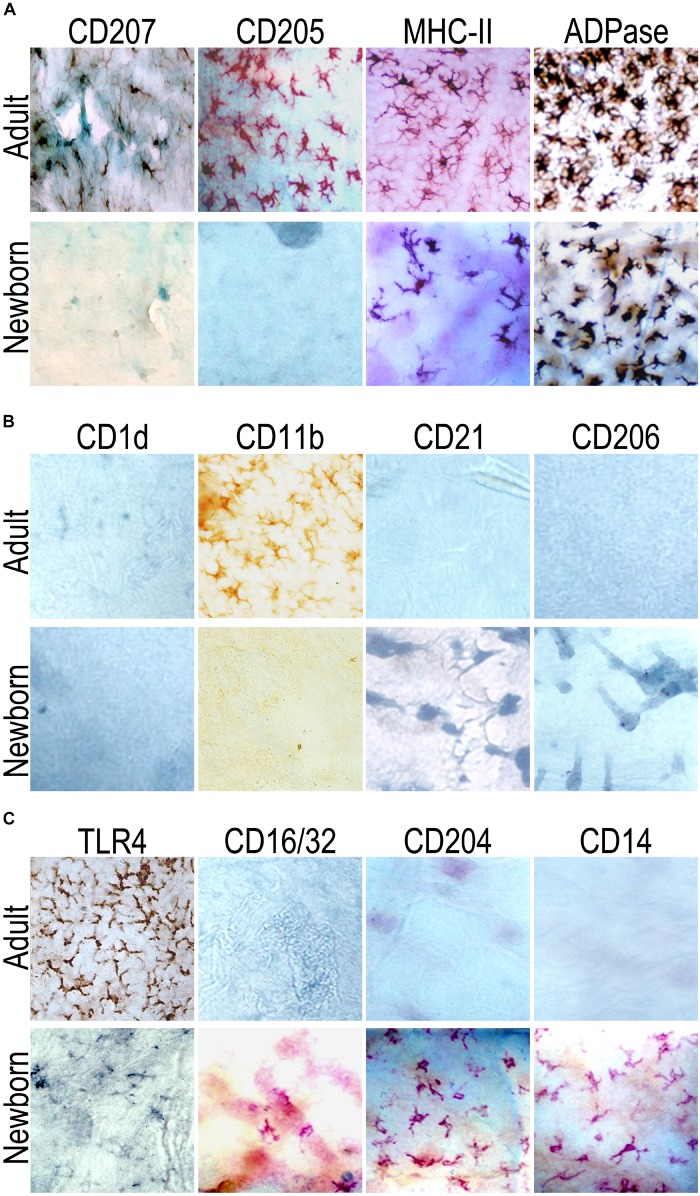
Langerhans cells (LC) in neonates can be identified by means of ADPase staining and express different molecules than in adults. Epidermal sheets from adult (top panel) or newborn (at birth, day 0, bottom panel) mice were probed for the expression of **(A)** CD207 (Langerin), CD205, MHC-II or ADPase activity. Neonatal epidermal LCs were positive for the ADPase activity and very few cells (in small clusters) expressed MHCII. **(B)** CD1d, CD11b, CD21, CD206; and **(C)** TLR4, CD16/32, CD204, and CD14. While neonatal epidermal LCs at birth were CD204+ and CD14+, epidermal LCs from adults did not express these molecules. Although the frequency of TLR4+ epidermal LCs in neonates was very scant, the TLR4+ cells in adults were widely distributed through the whole epidermis. Representative pictures are shown from at least nine mice evaluated in three independent experiments.

Initial attempts to label epidermal LCs from newborn (day 0) animals with markers typically present in adult LCs (Langerin (CD207), CD205) were unsuccessful. However, the presence of epidermal LCs was definitively revealed by the conspicuous, epidermal LC-restricted ADPase staining (33, 34). In fact, ADPase staining made evident that LCs in neonatal epidermis were as numerous as their adult counterparts. Besides this, it was possible to find MHCII + LCs in newborn mice, but these were very scarce and usually organized in small clusters (Figure 1A).

In order to evaluate *in situ* the phenotype of LCs in newborn and adult mice, we probed the epidermis for the expression of molecules putatively related with the function of antigen uptake or recognition, we thus included several putative PRRs or endocytic molecules: CD1d, CD206, TLR4; complement receptor molecules: CD11b and CD21; Fc-Receptors: CD16/CD32 and CD23 (data not shown); and CD14 and CD204 (Figures 1B,C).

This phenotypic analysis *in situ* revealed that in both newborn and adult mice there were TLR4 + LCs, whereas only in newborns there were CD16/CD32 + LCs. Interestingly, when we analyzed the expression of CD14 and CD204 in both adult and newborn mice, we could not observe positive cells for these molecules in epidermal sheets of adults. However, only in epidermal sheets of newborns (day 0) we found a network of CD14+ and CD204+ cells rather abundantly, which exhibited typical dendritic morphology. Except for CD11b detection in adult sheets only, we did not detect positive cells for CD1d, CD21, CD23 (data not shown) and CD206, neither in adult nor in newborn mice epidermis (Figures 1B,C). Evaluation of all these markers indicated us that LCs in neonatal skin express different molecules than those reported in adults.

### CD14+, CD204+ or TLR4+ Neonatal Epidermal Cells Are LCs

**FIGURE 2:**
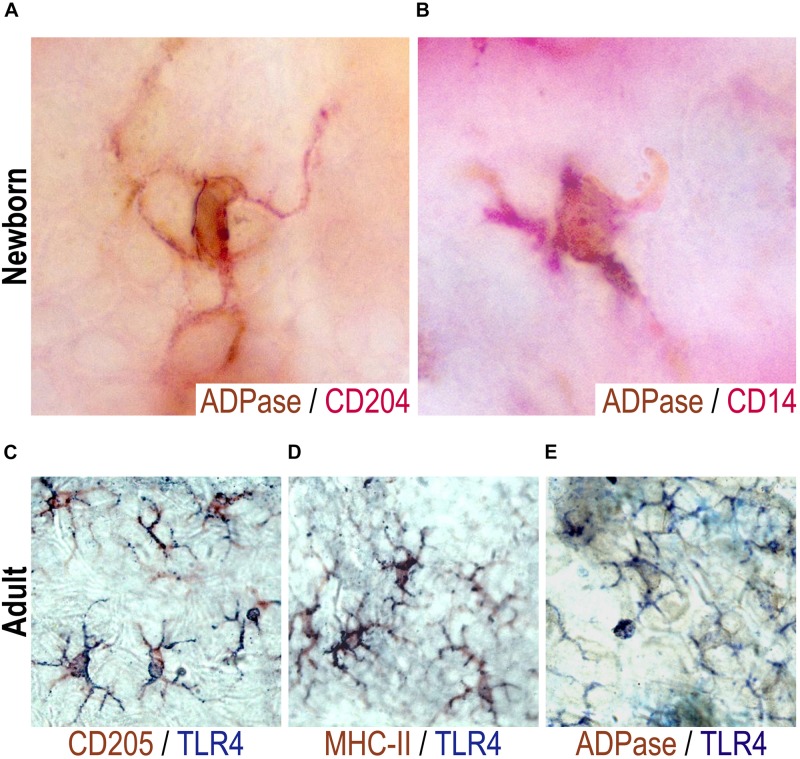
CD204+, CD14+, or TLR4+ neonatal epidermal cells are LCs. ADPase activity (brown) was determined together with a double staining with CD204 [red, **(A)**] or with CD14 [red, **(B)**] in epidermal sheets of mice at birth (day 0). Representative pictures are shown from at least two neonatal mice evaluated per experiment in three independent experiments. Double stainings were performed for TLR4 (blue) and CD205 [brown, **(C)**], MHC II [brown, **(D)**] or ADPase activity [brown, **(E)**] in epidermal sheets of adult mice. In adults, LCs were observed as TLR4+ADPase+, TLR4+DEC205+, TLR4+MHCII+. Representative pictures are shown from three adult mice evaluated per experiment in three independent experiments.

Some cells in the murine epidermis tend to adopt a rather dendritic morphology, LCs are characterized by the expression of MHCII, CD205, CD207, and ATPase/ADPase activity. Other populations such as dendritic epidermal T cells (DETC), express a canonical γδ TCR and the Thy molecule but lack typical LC markers, including ADPase, Langerin, CD205, among others. To determine whether CD14+ and CD204+ newborn epidermal cells are in fact LCs, double stainings were performed for ADPase and for CD14 or CD204 in epidermal sheets of newborn mice. LCs were observed as ADPase+/CD204+ (Figure 2A) or ADPase+/CD14+ (Figure 2B). Thus, we believe that the CD204+ and CD14+ epidermal cells exhibiting prominent dendritic morphology that we found in newborns, are indeed LCs.

We also found TLR4+ cells in both the adult and newborn epidermis. However, TLR4 seems not expressed in the whole cell in epidermal sheets of newborn mice and the frequency is very scant (Figure 1C), thus it was rather difficult to perform double labeling for TLR4 and ADPase activity in these neonatal mice. Due to this, we characterized TLR4+ cells with classic dendritic morphology in epidermal sheets of adult mice. Double stainings were performed for TLR4 and ADPase, CD205 or MHCII. LCs from adult mice were observed as CD205+/TLR4+ (Figure 2C), MHCII+/TLR4+ (Figure 2D) or ADPase+/TLR4+ (Figure 2E). Thus, adult TLR4+ epidermal LCs are indeed LCs. We thus inferred that TLR4+ cells in neonatal skin are also LCs.

### CD14+ and CD204+ Cells Seem to Disappear From Epidermis Rapidly After Birth, While CD205+ and CD207+ Cells Appear a Bit Later in the Neonatal Period

**FIGURE 3:**
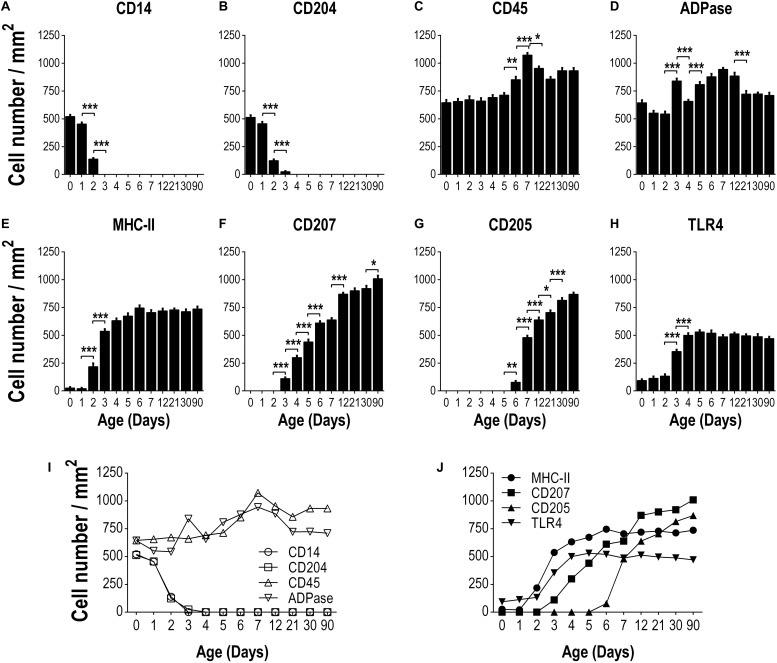
Kinetics of expression of different markers on epidermal LCs from the day of birth to adulthood. The frequency of cells positive for the following markers: CD14 **(A)**, CD204 **(B)**, CD45 **(C)**, ADPase **(D)**, MHCII **(E)**, CD207 **(F)**, CD205 **(G)** and TLR4 **(H)** was determined per area (mm^2^) of epidermal sheets, in at least ten fields for each sample, using at least three different epidermal sheets of the same animal. Data are from three independent where two to three mice were used per experiment and are expressed as the mean ± SD of cells/mm^2^ of epidermis. Integrated kinetic behavior of the markers from birth until adult age are shown in **(I,J)**. Data were analyzed by one-way ANOVA-Bonferroni. *p > 0.05, **p > 0.01, ***p > 0.001.

Interestingly, of the various markers evaluated, the only two endocytic molecules abundantly found in early neonatal (day 0) epidermal LCs were CDl4 and CD204, which somehow cover a wide spectrum of potential interactions with microorganisms. However, the *in vivo* functioning of both these molecules (CDl4 and CD204) remains to be ascertained in these neonatal LCs. We performed a kinetic follow-up of expression of these markers on LCs from birth to adulthood (counting LCs per mm^2^ on epidermal sheets at days 0, 1, 2, 3, 4, 5, 6, 7, 12, 21, 30, and 90). Noteworthy, at birth (on day 0), there is a striking coincidental expression between CDl4 and CD204 in epidermal LCs. Furthermore, the expression pattern follows an identical kinetic trend along days l, 2, and 3, until their complete disappearance by day 4 (Figures 3A,B), raising the possibility that these cells might be one and the same population. We also used CD45 to detect cells of hemopoietic origin throughout the study period. We observed both CD45+ and APDase+ cells *in situ* from birth to adulthood, indicative that these cells are leukocytes and a great frequency of those leukocytes are LCs (Figures 3C,D).

Regarding MHCII staining we observed an increase in the frequency of MHCII + LCs by day 2 of age (*P* < 0.05) reaching adult quantities by day 4 after birth (Figure 3E). Although CD205 and CD207 are expressed on adult epidermal LCs, we did not find these markers on epidermal sheets from newborn (day 0) mice (Figure 1A). Following the kinetic of expression, we detected CD207 + LCs by day 3 of age increasing gradually until reaching the adult frequency by day 12 of age (Figure 3F). On the other hand, CD205 + LCs were first seen by day 6 after birth, increasing dramatically from day 7 of age (*P* < 0.05) to adulthood (Figure 3G). The frequency of TLR4 + LCs increased by day 3 of age and reached the maximum frequency by day 4 of age (*p* < 0.05, Figure 3H). The integrated kinetic behavior of the markers assessed from birth to adulthood is shown in Figures 3I,J. Here, it can be clearly appreciated that the typical markers of LCs (MHCII, CD205, CD207) appear gradually after birth and newborns express molecules as CD14 and CD204 that disappear rapidly and are not found in adult skin.

### Neonatal Epidermal LCs Are Able to Take Up FITC as Cutaneous Antigen and Ferry It Into the DLNs

**FIGURE 4:**
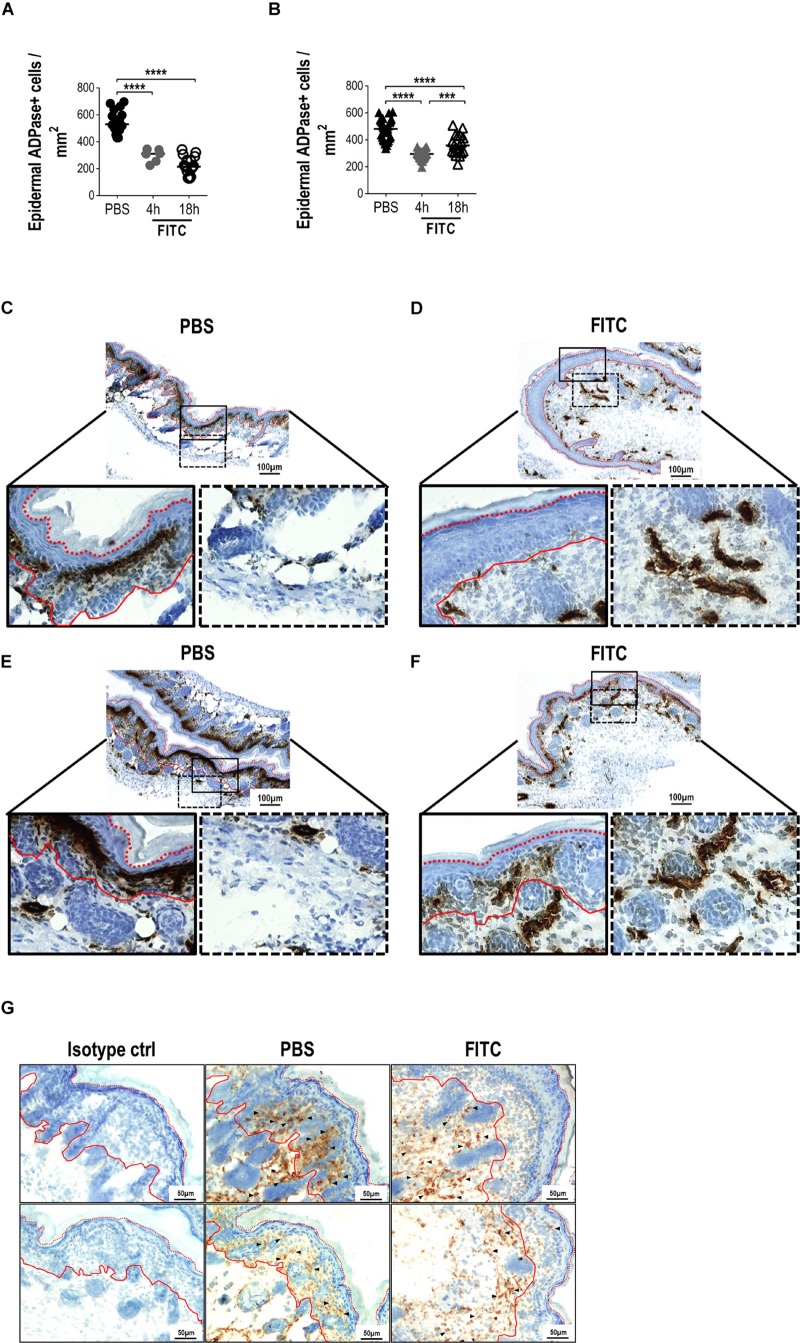
Analysis of epidermal sheets and skin cryosections from newborn mice 18 h after cutaneous FITC application. A solution of 0.5% FITC dissolved in 1:1 acetone:dibutylphthalate was applied topically over the skin of adult or newborn (day 0) mice. The frequencies of ADPase+ cells were determined per area (mm^2^) of epidermal sheets after 4 and 18 h of skin sensitization in adults **(A)** and neonates **(B)**. One dot represents the mean of five fields from one epidermal sheet section (2 × 3 mm). Three to six different random sections, where PBS or FITC was applied, were quantified per mouse. There were three independent experiments at 18 h and one experiment at 4 h where at least two adult mice and at least three neonates were used per condition. **(C–F)** Representative photomicrographs (magnifications: 10X and 40X) comparing the ADPase+ cells (dark brown) on cryosections from newborn mice after PBS [**(C,E)**, left panel] or FITC [**(D,F)**, right panel] application. Black lined squares represent 40X magnifications from epidermal (solid line) and dermal (dotted line) layers. (**C,E** and **D,F**) are replicates to show different mice and are representative of two independent experiments with five neonates per condition per experiment. **(G)** CD204 staining (DAB, brown) of neonatal skin cryosections (20X magnification). Two representative fields from PBS and FITC groups are shown from two independent experiments with five neonates per condition per experiment. Arrow heads shows some positive cells along the skin layers. Isotype control is shown in the left panel. Hematoxylin nuclear counterstaining is shown in blue. Red lines represent the limits of the basal (solid) and corneum (dotted) layers of the epidermis.

It is well documented that cutaneous hapten sensitization induces LCs migration from the skin to the DLNs and that this migration is associated with a concomitant increase of LCs into the DLN (35–37). To evaluate whether neonatal epidermal LCs were able to take up a cutaneous sensitizer, migrate and ferry it into the DLN, we topically applied FITC over the skin of both newborn and adult mice. We first evaluated the frequency of ADPase + LCs/mm^2^, 4 and 18 h after sensitization. As expected, the number of LCs decreased in the epidermis of sensitized adult mice at both times. The number of ADPase+ cells in the epidermis of newborn mice was also reduced showing a similar pattern than in adults (Figures 4A,B and Supplementary Figure S1). These results motivated us to look at skin cryosections to evaluate if LCs were migrating from epidermis to dermis in neonatal mice. We analyzed *in situ*, the frequency of ADPase+ cells 18 h after FITC cutaneous sensitization. We observed that most of the skin ADPase+ cells reside in the epidermis of control neonates (Figures 4C,E). However, the frequency of these ADPase+ epidermal cells decreased after 18 h of FITC sensitization while concomitantly increasing in the dermis (Figures 4D,F). Since we found that putative LCs express CD204 in neonate epidermis, we also evaluated the distribution of the CD204+ cells in skin cryosections after sensitization. The results showed a similar pattern compared to the ADPase+ cells. The frequency of CD204+ cells found in the epidermis after FITC application decreased compared to topical PBS while increasing in the dermis, a deep layer in the skin (Figure 4G). These results show that epidermal LCs in neonates are indeed migrating out from the epidermis into the dermis.

**FIGURE 5:**
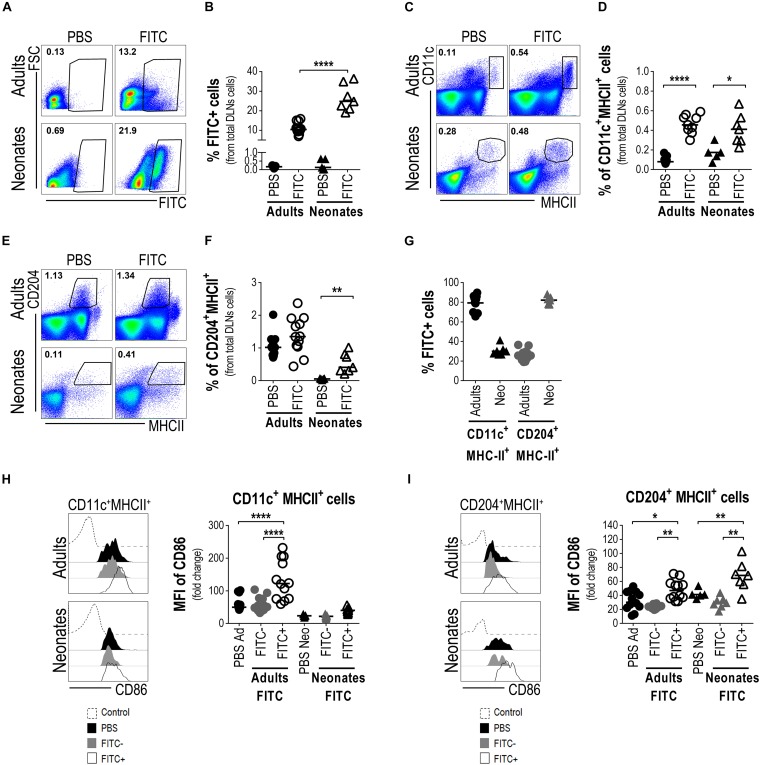
Neonatal epidermal LCs are able to take up FITC as cutaneous antigen and ferry it into the draining lymph nodes. Draining lymph nodes from FITC-sensitized skin obtained from newborn and adult mice were removed 18 h after cutaneous sensitization. Representative plots **(A)** and frequency **(B)** of FITC+ cells from DLNs suspensions were evaluated by FACS. The frequencies of CD11c+MHCII+ cells **(C,D)** and CD204+MHCII+ cells **(E,F)** were determined by FACS in the FITC-sensitized skin-DLNs. **(G)** The frequency of FITC+ cells from CD11c+MHCII+ and CD204+MHCII+ cells was analyzed in the DLNs of adult and newborn mice 18 h after cutaneous FITC application. **(H,I)** The expression levels of CD86 were calculated as fold increase of the Mean Fluorescence Intensity (MFI) of CD86 between the CD11c+MHCII+ **(H)** or the CD204+MHCII+ **(I)** cells expressing CD86+ and the non-staining control. Representative histograms show expression levels of CD86 on the indicated populations. FITC-CD11c+MHCII+ and FITC-CD204+MHC+cells from FITC sensitized mice were also used as internal/comparative controls. Likewise, newborn and adult mice receiving only PBS were used as controls for all the experiments. In FACS analysis, subpopulations were gated from Singlets/Lymphocytes/Live cells (Supplementary Figure S2). Dots represent independent samples and horizontal line indicates the median and were analyzed by one-way ANOVA-Bonferroni. *p < 0.05, **p < 0.01, ****p < 0.0001. Samples were pool of brachial, axillary and inguinal lymph nodes from six to eleven pups per offspring and are from at least three independent experiments.

Then, to evaluate if these LCs were able to reach the skin-DLNs, we analysed by FACS the presence of FITC+ cells in neonates’ and adults’ DLNs (from total lymphocytes, Supplementary Figure S2). As expected and as established in adult mice (35), FITC+ cells were found in DLNs suspensions. Interestingly we also found a FITC+ population in neonatal DLNs and the proportion of these cells was relatively higher, compared to adults (Figures 5A,B). We also evaluated the proportion of CD11c+MHCII++ within DLNs from both adult and neonatal mice 18 h after cutaneous FITC application. The proportion of CD11c+MHCII++ cells increased significantly in the DLNs of adult mice 18 h after skin sensitization. Although the CD11c+MHCII++ cell population of newborn mice is not as clear as the one in adults, we gated on CD11c+MHCII+ and observed a slight increase in the proportion of this population compared with the PBS control (Figures 5C,D). As MHCII is an important molecule for APCs, and since we found an abundant network of CD204 + LCs in neonate epidermis, we looked for CD204+MHCII+ cells in the DLN. In contrast to adults where the proportion of CD204+MHCII+ cells in the DLNs did not change after 18 h of topical FITC compared to PBS controls, the frequency of CD204+MHCII+ cells in the DLNs of newborn mice increased significantly (*p* < 0.01) 18 h after cutaneous FITC application, compared to PBS (Figures 5E,F). The CD11c+ and CD204+ cell populations are clearly different in their MHCII expression in adult mice but this seems not to be the case in neonates. As both the CD11c+ and the CD204+ populations were gated together with the expression of MHCII, we investigated whether these cells would represent the same population. To this end, we analyzed both molecules by gating on CD11c vs. CD204 and we found they are two different subpopulations (Supplementary Figure S3). Then, we evaluated the presence of FITC within the CD11c+MHC+ and CD204+MHC+ subpopulations in DLNs by FACS. In DLNs from adult mice topically sensitized with FITC, the CD11c+MHCII+ subpopulation was the main one containing FITC. In contrast, in DLNs from newborn mice sensitized with FITC the result was quite different being mainly the CD204+MHCII+ subpopulation the one that contains the FITC (Figure 5G). Finally, we evaluated the expression of the co-stimulatory molecule CD86 in both the CD11c+MHC+ and the CD204+MHC+ subpopulations. The CD11c+MHC+FITC+ fraction in adult mice sensitized with FITC increased significantly the expression of CD86 within the DLNs (*p* < 0.0001, Figure 5H) and to a lesser extent the CD204+MHC+FITC+ subpopulation (*p* < 0.05, Figure 5I) compared to PBS control mice and the FITC- fractions. In neonates sensitized with FITC, we did not see big differences in the intensity of CD86 expression within the CD11c+MHCII+FITC+ subpopulation compared to the FITC negative- fraction and the PBS control mice (Figure 5H). Interestingly, the CD204+MHCII+FITC+ cells within DLNs from FITC-sensitized newborn mice increased significantly the expression of CD86 compared to the FITC negative-fraction and the PBS control mice (*p* < 0.01, Figure 5I).

Altogether, these findings strongly point to neonatal epidermal CD204 + LCs as the ones that are able to take up FITC, to increase co-stimulatory molecules and to carry the FITC into the lymph nodes draining the sensitized skin, and also suggest their function as APCs.

### Neonatal T Cells Can Be Activated and Induced to Proliferation Within the Regional Draining Nodes *in vivo*

**FIGURE 6:**
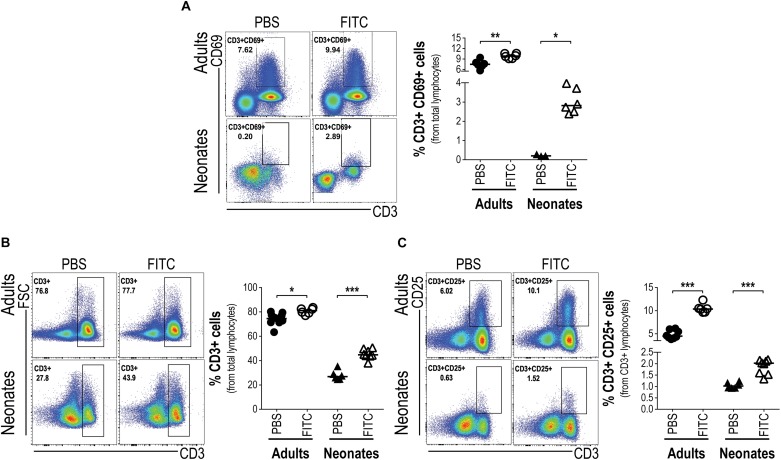
Upon antigen delivery into DLNs neonatal T cells increase percentages and respond with activation. Skin-draining lymph nodes from FITC-sensitized newborn and adult mice were removed 18 and 72 h after topical FITC application, and analyzed by FACS. The expression of the early activation marker CD69 was determined on CD3+ T cells 18 h after cutaneous FITC application **(A)**. The percentages of DLN CD3+ **(B)** and CD3+CD25+ **(C)** T cells were analyzed 72 h post-cutaneous application of FITC. In FACS analysis, subpopulations were gated from Singlets/Lymphocytes/Live cells. Dots represent independent samples and horizontal line indicates the median and were analyzed with Mann-Whitney *U* test, *P < 0.05; **P < 0.01, ***p < 0.001. Samples were pool of brachial, axillary and inguinal lymph nodes from six to eleven pups per offspring and are from at least three independent experiments.

As neonatal epidermal LCs seem able to take up cutaneous FITC, to ferry it into the DLN and become activated, we wanted to know whether these cutaneous LCs could activate the neonates’ T cells within DLNs. To this end, we evaluated in DLNs the frequency of CD3+ T cells which were expressing the activation marker CD69 18 h post-topical FITC application. Interestingly enough, neonates receiving topical FITC increased in DLNs the frequency of CD3+CD69+ T cells 13 fold (*p* < 0.05) compared to topical PBS. In the case of adult mice, as expected, the frequency of CD3+CD69+ T cells was higher after 18 h after topical FITC compared to PBS application, however, the increase was relatively smaller than that seen in neonate mice (Figure 6A). This early activation of T cells after 18 h of FITC application prompted us to evaluate the proliferation of those T cells inside DLNs. As a first clue we found that the frequency of CD3+ T cells increased about twice in neonates 48 and 72 h after FITC application (Figure 6B and Supplementary Figure S4). This correlated well with a higher frequency of CD3 + T expressing CD25 (Figure 6C and Supplementary Figure S4), the high-affinity receptor for IL-2, the T cell inducing-proliferation cytokine (38). As expected, CD3+ T cells and CD3+CD25+ T cells also increased in adult mice after 48 and 72 h of FITC application compared to PBS controls, however, the extent of these increases were lower than in neonates, especially in CD3+ T cells (Figures 6B,C and Supplementary Figure S4).

**FIGURE 7:**
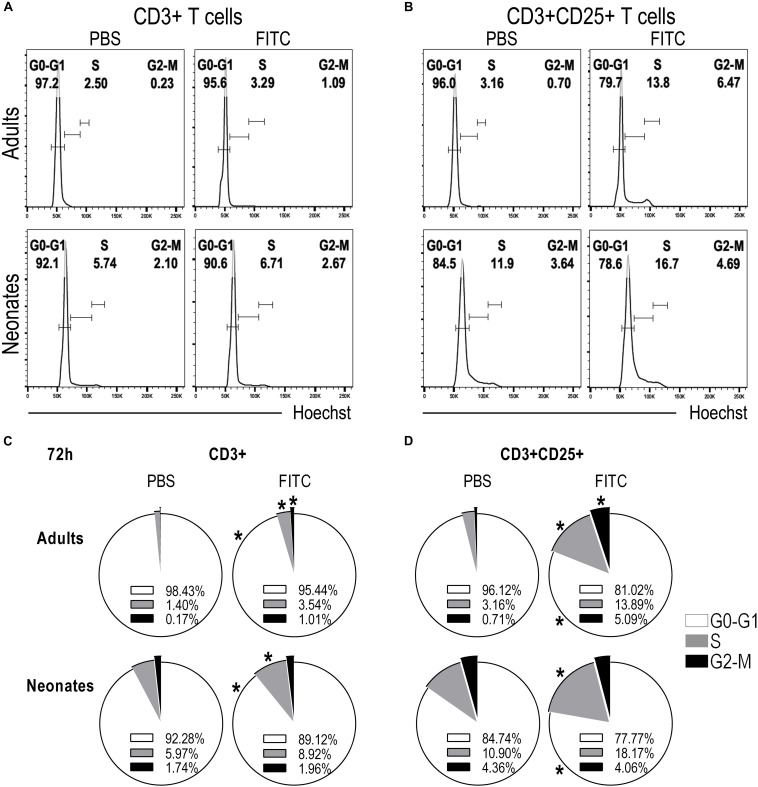
Neonatal T cells are able to proliferate upon antigen delivery into DLNs. Skin-draining lymph nodes from FITC-sensitized newborn and adult mice were removed 72 h after topical FITC sensitization, and analyzed by FACS. To evaluate whether T cells were able to start proliferating, we assessed the different phases of the cell cycle by staining the cells with Hoechst 33342 and analyzing DNA content, 2n for G0-G1 phase, 4n for G2-M and cells in S-phase were found with a DNA content ranging between 2n and 4n. **(A,B)** Histograms of DNA content against cell numbers give the typical DNA profile of proliferating cells for CD3+ **(A)** and CD3+CD25+ **(B)** T cells. **(C,D)** Pie charts summarize cell cycle profiles (at 72 h) as percentage of cells in G0-G1 Phase (white), in S Phase (gray), and in G2-M Phase (black) on CD3+ **(C)** or CD3+CD25+ **(D)** T cells. Data represent the mean and were analyzed with Mann-Whitney *U* test, *P < 0.05 FITC vs. PBS on neonates or adults, respectively. Samples were pool of brachial, axillary and inguinal lymph nodes from six to eleven pups per offspring and are from at least three independent experiments.

To further substantiate that T cells were indeed proliferating we evaluated in DLNs the phases of the cell cycle of CD3+ and CD3+CD25+ T cells after 48 (Supplementary Figure S5) and 72 h post-topical application of FITC or PBS. We found neonatal CD3+ T cells on S phase of the cell cycle increased compared to CD3+ T cells from animals that received PBS only. This raise in S phase correlated with the decreased frequency of CD3+ T cells in G0-G1 phase and the slightly increased frequency of CD3+ T cells in G2-M phase (Figures 7A,C). The increase of cells in S phase and the decrease of cells in G0-G1 phase of the cell cycle in neonates were more evident in the CD3+CD25+ T cell subset (Figures 7B,D).

These clear evidences of neonatal T cells responding inside DLNs, together with the arrival of CD204+FITC+ putative epidermal LC upregulating expression of MHC and CD86 in DLNs, are highly suggestive that neonatal epidermal LCs are indeed capable of accomplishing all these tasks; to take up a cutaneous sensitizer, to ferry it into DLNs and to activate neonatal T cells *in vivo*.

## Discussion

Our study focused on the ontogenic and functional development of LC in early neonates, especially regarding the potential capacity of these LCs to carry cutaneous antigens from the skin to DLNs and to activate neonatal T cells *in vivo*.

We assessed the LC network in the epidermis of newborn mice in their native unperturbed state during the early neonatal period by staining for ADPase and CD45, very few of them expressed MHCII (usually grouped in small clusters) or TLR4. Further phenotype characterization revealed that they acquired CD205 and CD207 rather abruptly after a few days post-birth, as it has been shown before (29, 39). We observed an increase in the frequency of MHCII+ cells at day 3 after birth, which concurs with some increase of ADPase + LCs. Although we could detect CD11b on LCs from adult animals, as described before (35, 40), we were not able to find its expression on epidermal sheets from neonates, even when trying different fixation approaches. Several studies have reported that murine fetal epidermis contains ADPase+CD11b+F4/80+CD32+CD115+CX3CR1+MHCII− CD205− CD207− CD90− CD3− dendritic epidermal leukocytes, distinct mice strains may have an impact on these differences (25, 26, 29, 41, 42). Nowadays we know that primitive myeloid LC progenitors can be detected around embryonic day 18 and derive predominantly from fetal liver monocytes, these precursors actively proliferate in the skin and acquire MHCII, CD11c and Langerin after the first week of birth (27–29). Major transcriptomic changes occur after postnatal period and give them the LC identity (43). Although duality has been suggested in the origin of LCs, as it has been shown that they express Zbtb46 which is characteristic of DCs; while they originate from progenitors that express Mafb indicating their macrophage origin (44), evidence support a macrophage origin (45). To the best of our knowledge, our study seems to be the first to show that epidermal LCs express TLR4 *in situ* in both adult and neonatal mice. It has been shown before the low expression of mRNA of *tlr4* but not the protein itself (17). Furthermore, we found that ADPase + LCs in neonates at birth are CD204 + and CD14 + , markers which were both absent in adult mice. Since at birth LCs do not express Langerin, the positive staining with ATP/ADPase provides compelling evidence that ADPase+ cells in epidermis are bona fide LCs (33, 34, 41). It is particularly intriguing the rapid apparent disappearance of CDl4 and CD204 within the narrow window of the first three days of birth, maybe its function is compensated by the concomitant increase of TLR4 and MHCII expression, and the appearance of CD205 and CD207. The uninterrupted ADPase- and CD45-positivity along this neonatal period is indicative of the continuous presence of LCs and rather suggests a potential down/up-regulation of certain markers. It is worth to mention that CD14 + APCs have been described in human skin during the fetal stage and that these cells in the dermis constitute a tissue-resident population with a short half-life of <6 days (46, 47). With our results we now add information regarding the postnatal down/up-regulation of LC markers during the first week of life. Notably, all these changes fit well with LC differentiation kinetics from monocytic precursors shown in other studies (27, 45). One limitation of our work is that all data collected are from BALB/c mice. However, taking in consideration that others have described LCs precursors are present even on embryonic day 18 either on BALB/c or C57BL/6 animals and that MHCII and CD207 are expressed from day 3 post-birth (25–27, 29, 30); we may assume that neonatal LCs might differ from the expression of adult markers for other strains too.

Migration of LCs from the skin to DLNs after topical FITC suggests that LCs in newborn mice can respond to a local stimulus, indicative of their functional capacities. However, much more work is needed to understand the early maturation and differentiation of LC *in vivo*. Whether the rapid shifting of these putative LCs away from the neonatal phenotype is autonomously programmed, dependent upon a still undefined local microenvironment, or linked to exogenous environmental signals, is still unknown to us, but these topics certainly merit attention to better understand the early ontogeny of immune responses, both innate and adaptive.

An important feature of the processes in which LCs are involved in health and disease is their ability to migrate to the DLN and interact with T-cells. LCs migrate from epidermis in response to wide diversity of stimuli, including microbes or their products, UV exposure or chemical sensitizers (35–37). Among others, CHS response has been used extensively to understand some of the biological functions of LCs. However, nowadays the role of LCs in the CHS is controversial given that haptens are small molecules that can reach not only the epidermis but also the dermis (48–51). It has been seen that depletion of LCs using Langerin-DTR mice does not completely nullify the CHS responses, which implies the potential involvement of other skin APCs (52, 53). However, as LCs absence induces enhanced CHS, this also implies that LCs do have a role during CHS responses. It is conceivable that differences in responses to chemical sensitizers depend on the type of sensitizer, the dose and the experimental model used (48–51). Most of these studies have been conducted extensively in adult animals whereas much less is known about the role of LCs in neonates, especially the functioning at very early ages where phenotypic changes might be drastic and rapid, moreover, what little is known can be controversial. Previously Dewar et al., showed that LC from 3-day-old newborn mice were inefficient to ferrying FITC to the lymph nodes and displayed a reduced CHS response against trinitrochlorobenzene (39) while more recently Flores-Maldonado et al., has shown that newborn mice were able to elicit an adaptive cell-mediated immunity against *Candida albicans* inoculated epicutaneously at birth, which was demonstrable by a delayed-type hypersensitivity response when challenged in adulthood (54). Our results concur with the latter and show that epidermal LCs from newborn (day 0) mice can take up a cutaneous sensitizer *in vivo* and perform the ensuing functions that adult LCs do. This is reinforced by the subsequent appearance in skin-DLNs of a CD204+MHC+ population containing FITC. Notably, these cells significantly up-regulated CD86, compared to the CD11c+MHC+ cells in neonates. In contrast, in adult DLNs the main population containing FITC was the CD11c+MHC+. Altogether, these results strongly point to neonatal epidermal CD204 + LCs as the ones that are able to take up FITC *in vivo* and to carry it into the skin-draining DLNs. Also this suggests that these neonatal LCs might already act as competent APCs at these early times of life. However, further studies are required using different haptens and other experimental models.

A key aspect of the proper functioning of APCs is the initiation of the adaptive immune responses, especially the activation and proliferation of T cells. It has been previously demonstrated in adults that in addition to the stimulation of the TCR and costimulatory molecules, γc cytokines such as IL-4 and IL-2 promote proliferation (38, 55–57). Importantly, we found in DLNs that neonatal T cells are activated early after topical FITC, demonstrated by the up-regulation of CD69 18 h after cutaneous FITC. Through cell cycle assessments, we further confirmed that the initial activation of neonatal T cells led them to proliferation. These latter findings imply that T cells from newborn mice are indeed capable to respond *in vivo* to the cutaneous-derived signals, a topic that has been re-discussed recently (10, 58).

Our study shows that bona fide LCs in the epidermis of mice at birth are as abundant as those in adults, but differentially express surface receptors. These cells lack CD205, CD207 and express low levels of MHCII and TLR4 molecules at birth. Interestingly, these putative LCs express other molecules such as CD204 and CD14. Whether these cells that rather abruptly downregulate CDl4 and CD204 within days l–3 of extrauterine life constitute exactly the same population changing phenotype at later days, is still unknown, careful tracing experiments could help us to clarify this. To the best of our knowledge, this study is the first to show TLR4 on LCs *in situ* in adult and neonatal mice. Our data strongly suggest that *in vivo* neonatal epidermal LCs are endowed with the capacity to take up a cutaneous antigen, to get activated and to carry this antigen toward T cells into DLNs, and last but not least, that neonatal T cells can respond with activation and proliferation *in vivo*.

## Data Availability Statement

All datasets generated for this study are included in the article/Supplementary Material.

## Ethics Statement

The animal study was reviewed and approved by the Animal Use Guidelines for animal care and experimentation UPEAL-CINVESTAV-IPN. All procedures were reviewed and approved by the UPEAL-CINVESTAV Ethics Review Committee.

## Author Contributions

MB-G, JY-P, RM-A, and LF-R conceived and designed the experiments, analyzed the data, and interpretation and discussion. MB-G, JY-P, RM-A, NB-C, MH-C, IG-H, JC-A, RM-F, LD-M, and AF-L performed the experiments and the acquisition of data. MB-G, JY-P, RM-A, NB-C, and LF-R wrote and revised the manuscript. All authors approved the final version to be published.

## Dedication

In memoriam of Professor Leopoldo Flores-Romo, a brilliant Mexican immunologist who inspired through his passion to many scientists generations. His legacy will last forever.

## Conflict of Interest

The authors declare that the research was conducted in the absence of any commercial or financial relationships that could be construed as a potential conflict of interest.

## Supplementary Material

FIGURE S1 ADPase activity analysis of epidermal sheets from newborn mice 4 and 18 h after cutaneous FITC application. Representative micrographs (10X magnification) of ADPase + cells from neonatal epidermal sheets after PBS or 4 and 18 h of FITC skin sensitization. PBS group was used as control.

FIGURE S2 Strategy used for flow cytometry analysis. Dot plots show the gating strategy used to identify adult and newborn subpopulations from DLNs. According to singlets, forward and side scatter characteristics (FSC and SSC), live cells were assessed for the expression of CD11c or CD204 and the simultaneous positivity of MHCII + staining. At least 750 × 10^3^ cells were acquired per condition.

FIGURE S3 Analysis of CD11c and CD204 co-expression in DLNs suspensions from neonates. The co-expression of CD11c and CD204 was evaluated on single/live cells from DLNs of neonates. Samples from skin-sensitized FITC and PBS control mice show that CD11c+ and CD204+ cells apparently represent two different subpopulations.

FIGURE S4 T cell activation in skin-draining lymph nodes after 48 h of topical application of FITC. Skin-draining lymph nodes from FITC-sensitized newborn and adult mice were removed 48 h after topical sensitization, and analyzed by FACS. The percentages of CD3+ and CD3+CD25+ T cells were analyzed. Subpopulations were gated from Singlets/Lymphocytes/Live cells. Dots represent independent samples and horizontal lines indicate the median and were analyzed with Mann-Whitney *U* test, **P* < 0.05; ***P* < 0.01. Samples were pool of brachial, axillary and inguinal lymph nodes from six to eleven pups per offspring and are from at least three independent experiments.

FIGURE S5 Cell cycle analysis of T cells from skin-draining lymph nodes after 48 h of topical application of FITC. Skin-draining lymph nodes from FITC-sensitized newborn and adult mice were removed 48 h after topical sensitization, and analysed by FACS. Pie charts summarize cell cycle profiles as percentage of cells in G0-G1 Phase (white), in S Phase (gray), and in G2-M Phase (black) of CD3 + (C) or CD3 + CD25 + (D) T cells. Data represent the mean and were analysed with Mann-Whitney *U* test, **P* < 0.05 FITC vs. PBS on neonates or adults, respectively. Samples were pool of brachial, axillary and inguinal lymph nodes from six to eleven pups per offspring and are from at least three independent experiments.

## Abbreviations

APC: antigen presenting cell
CSH: contact hypersensitivity
DLN: draining lymph node
FITC: fluorescein isothiocyanate
LC: langerhans cell
PRR: pattern recognition receptor
TLR: toll-like receptor.
