# Supplementary material for: Langerhans Cells From Mice at Birth Express Endocytic- and Pattern Recognition-Receptors, Migrate to Draining Lymph Nodes Ferrying Antigen and Activate Neonatal T Cells in vivo
Source: Front Immunol. 2020 Apr 27;11:744. doi: 10.3389/fimmu.2020.00744 (PMC7197463; doi:10.3389/fimmu.2020.00744)

Langerhans cells from mice at birth express endocytic- and pattern recognition-receptors, migrate to draining lymph nodes ferrying antigen, and activate neonatal T cells *in vivo*

Becerril-García MA[1, 2, 5], Yam-Puc JC[1, 3, 5], Maqueda-Alfaro RA[1], Beristain-Covarrubias N[3], Heras-Chavarría M[1], Gallegos-Hernández IA[1], Calderón-Amador J[1], Munguía-Fuentes R[1], Donis-Maturano L[1, 4], Flores-Langarica A[3], Flores-Romo L[1*].

Supp Figure 1. ADPase activity analysis of epidermal sheets from newborn mice 4h and 18h after cutaneous FITC application.

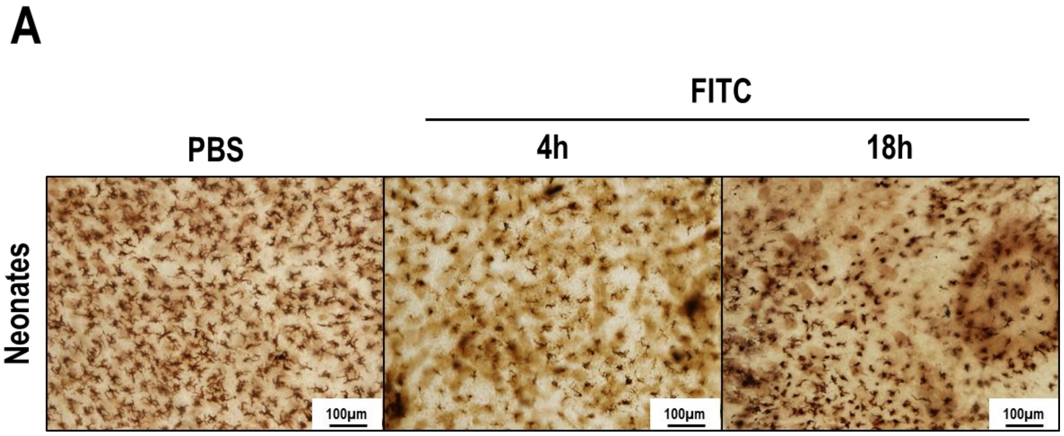

Supp Figure 2. Strategy used for flow cytometry analysis.

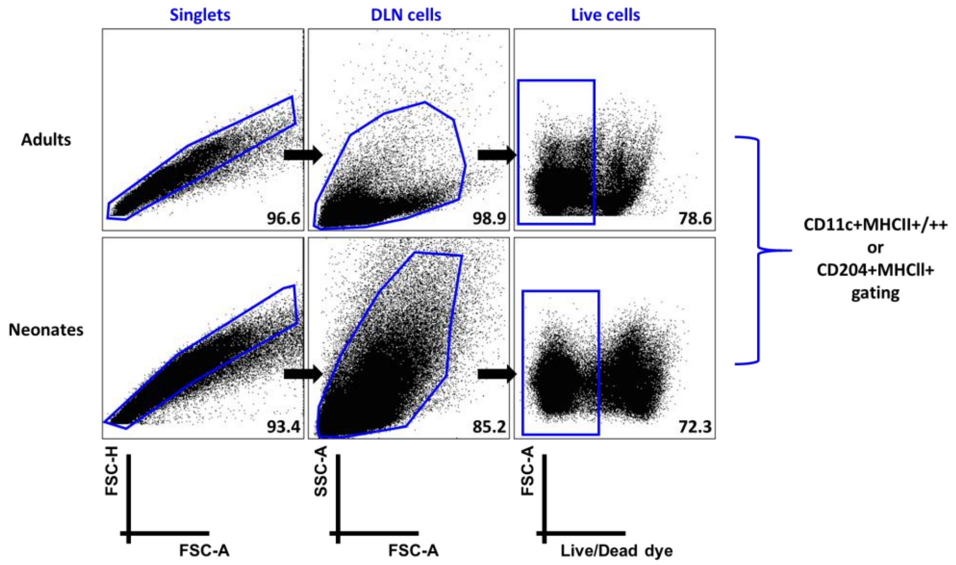

Supp Figure 3. Analysis of CD11c and CD204 co-expression in DLNs suspensions from neonates.

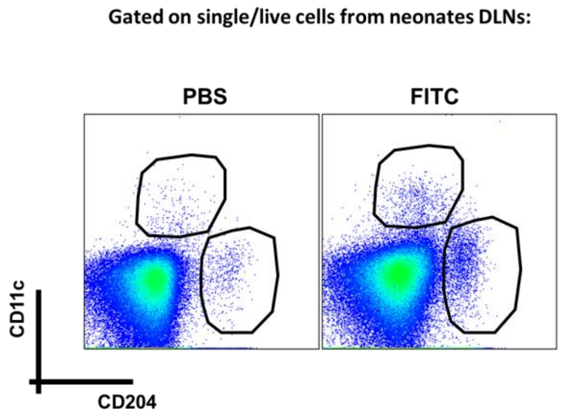

Supp Figure 4. T cell activation in skin-draining lymph nodes after 48h of topical application of FITC.

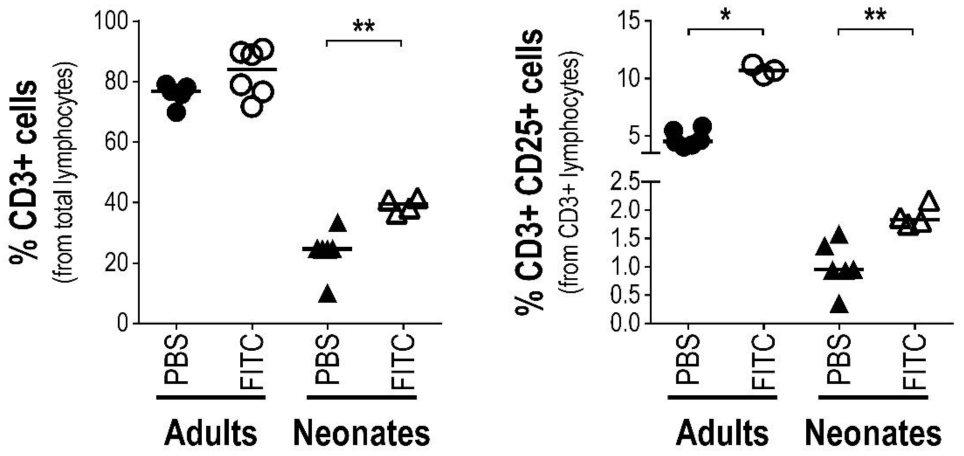

Supp Figure 5. Cell cycle analysis of T cells from skin-draining lymph nodes after 48h of topical application of FITC.

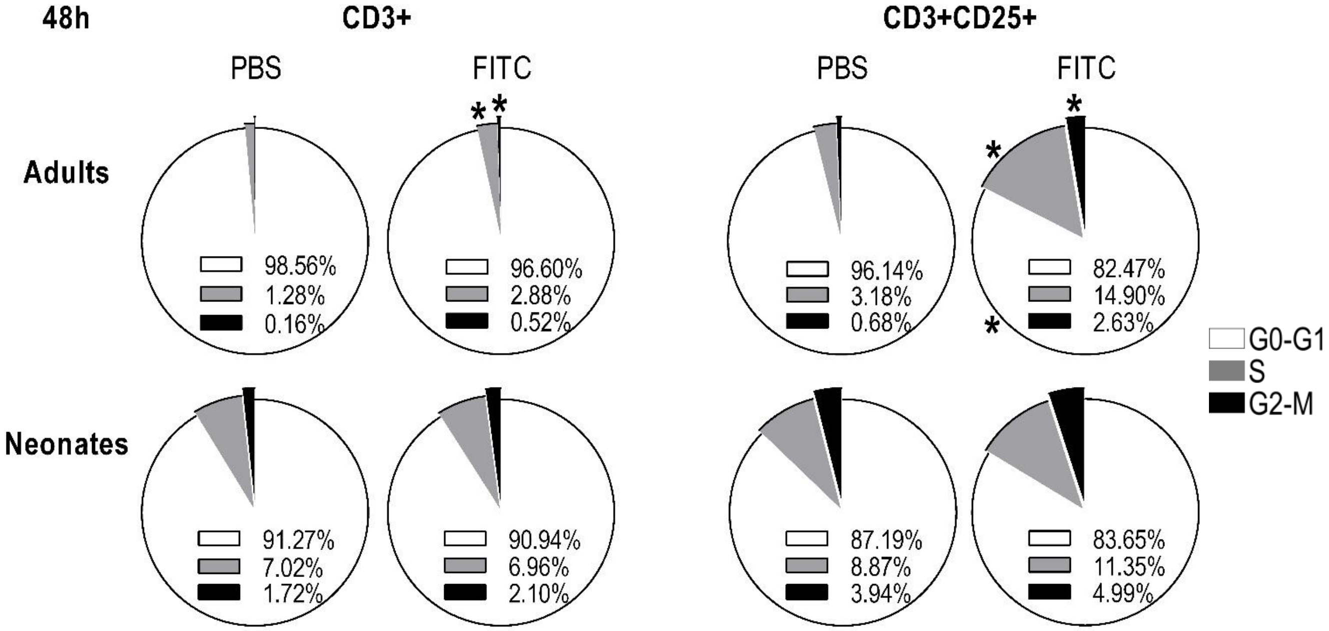

Supplement: FIGURE S1 — ADPase activity analysis of epidermal sheets from newborn mice 4 and 18 h after cutaneous FITC application. Representative micrographs (10X magnification) of ADPase + cells from neonatal epidermal sheets after PBS or 4 and 18 h of FITC skin sensitization. PBS group was used as control. [file Data_Sheet_1.PDF]
